# The impact of the lung environment on macrophage development, activation and function: diversity in the face of adversity

**DOI:** 10.1038/s41385-021-00480-w

**Published:** 2022-01-11

**Authors:** Calum C. Bain, Andrew S. MacDonald

**Affiliations:** 1 The University of Edinburgh Centre for Inflammation Research, Queen’s Medical Research Institute, Edinburgh Bioquarter, Edinburgh, EH16 4TJ UK; 2 Lydia Becker Institute of Immunology and Inflammation, University of Manchester, Manchester, M13 9NT UK

## Abstract

The last decade has been somewhat of a renaissance period for the field of macrophage biology. This renewed interest, combined with the advent of new technologies and development of novel model systems to assess different facets of macrophage biology, has led to major advances in our understanding of the diverse roles macrophages play in health, inflammation, infection and repair, and the dominance of tissue environments in influencing all of these areas. Here, we discuss recent developments in our understanding of lung macrophage heterogeneity, ontogeny, metabolism and function in the context of health and disease, and highlight core conceptual advances and key unanswered questions that we believe should be focus of work in the coming years.

## Introduction

Macrophages are largely sessile, tissue dwelling phagocytes that are present in every organ of the body. The biology of macrophages has been studied for well over one hundred years since being described by Elie Metchnikoff at the end of the nineteenth century. However, arguably, it is the last 10–15 years that has seen major revisions of key concepts in macrophage biology, from nomenclature of subsets and activation states to the ontogeny of these cells. For instance, while macrophages have historically been described as M1 (“classically-activated”) or M2 (“alternatively-activated”) largely based on in vitro culture systems^1^, it is now beyond doubt that a binary classification such as this is inadequate to capture the complexity of macrophage plasticity and activation states, particularly in vivo^2^. Technological advances have revealed tremendous diversity and heterogeneity between macrophages from different tissues and even within different niches of the same tissue in terms of phenotype, transcriptome and metabolome^3,4^. While macrophages generally excel at the ‘silent’ clearance of debris, apoptotic host cells and the capture and destruction of microbial intruders, it is now clear they also play far broader roles and are exquisitely tailored to meet the demands of the local tissue microenvironment in which they reside. Indeed, the in vivo tissue environment may be the major determinant governing macrophage development, recruitment, activation and function, highlighting the importance of careful consideration of the distinctive properties that different tissues possess, in steady state and during inflammation, to fully understand the role of macrophages in different locations throughout the body.

In this article, we review recent developments in the understanding of macrophage heterogeneity, ontogeny and function in lung health and during inflammation, immunity and tissue repair. Using pulmonary fibrosis as an example, we discuss how dysregulated macrophage behaviour can contribute to lung pathology and how mouse models have revealed the complexity of the macrophage response to lung injury. Along the way we identify key areas that we believe warrant further investigation.

## Defining pulmonary macrophages in the healthy lung

Macrophages are distributed throughout the lung and can broadly be divided into those present in the airways/alveoli and those in the tissue interstitium/parenchyma. While it has been clear for decades that pulmonary macrophages exist in both airways and tissues, it is only recently that we have come to appreciate the degree of heterogeneity and diversity between different macrophage subsets in each location. In particular, the advent of single cell technologies, such as single cell RNA sequencing (scRNA-seq), has allowed tissue macrophage heterogeneity to be assessed in a completely unbiased manner across several species^5–11^.

In mice, alveolar macrophages (AlvMϕs; see Box 1) in the healthy lung are defined by their high and uniform expression of CD11c, SiglecF and CD169 (Siglec1; sialoadhesin), and lack of CD11b expression^12–14^. Their residence in the airways can be confirmed by performing bronchoalveolar lavage (BAL) where they are the sole macrophage population in health^15^, although it is important to note that this method only retrieves a fraction of the macrophages resident in the airways. Murine interstitial macrophages (IntMϕs) express high levels of CD11b, but lack expression of SiglecF^12–14^. Compared with AlvMϕs, considerably less is known about the IntMϕ compartment, likely reflecting the fact that these macrophages are difficult to isolate from lung tissue using standard enzymatic protocols. Indeed, flow cytometric analysis of whole mouse lung digests suggests that IntMϕs form a small fraction of the overall macrophage compartment in health, with AlvMϕs outnumbering them by 5–10-fold^15,16^. Whether this is also the case in humans is currently hard to determine with certainty, as no comparable set of surface markers has yet been identified to unequivocally distinguish human AlvMϕs from IntMϕs in BAL, sputum or in lung tissue digests. Nevertheless, limited fluorescence microscopy of mouse and human lung, which circumvents the need to dissociate solid tissue, suggests that tissue (IntMϕs) are more abundant than suggested by flow cytometry^17–19^. Thus, the notion that these cells represent a minor macrophage population should be reconsidered (Box 2).

**Fig. 1:**
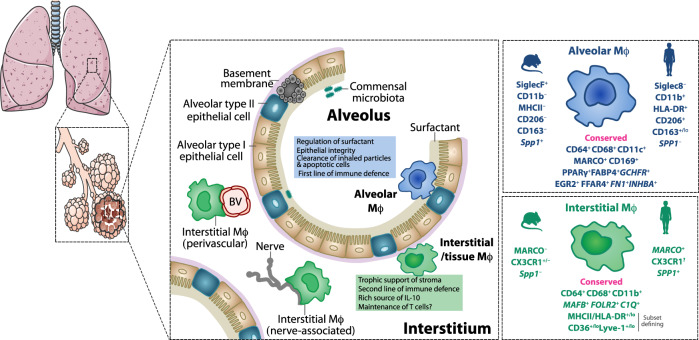
Heterogeneity, phenotypic profiles and functions of macrophages in the healthy lung. The lung macrophage compartment is heterogeneous, with at least two populations occupying distinct anatomical niches in the healthy lung. Macrophages are present in the bronchoalveolar space, including the alveoli where gaseous exchange occurs. Alveolar macrophages (AlvMϕs) are defined by their expression of CD11c, MARCO and CD169 in both mice and humans, although additional species-specific markers must be used to define them accurately. AlvMϕs are crucial for regulating surfactant produced by the respiratory epithelium as well as maintaining epithelial integrity and responsiveness. Their high phagocytic capability allows them to clear apoptotic/senescent cells and inhaled particles efficiently. They also act as the first line of defence against air-borne pathogens, although the relative role of resident AlvMϕs versus elicited, monocyte-derived macrophages in immune protection varies depending on the nature of the insult (see text and Fig. 3). Macrophages are also found in the interstitial space between the alveoli and the capillary beds, as well as surrounding larger airways (bronchi). These interstitial macrophages (IntMϕs) are phenotypically distinct from AlvMϕs and at least two subsets exist in mouse and man defined by differential expression of MHCII (HLA-DR), Lyve-1 and/or CD36. IntMϕs may act as a second line of defence under the epithelial barrier and basement membrane. In health, they may support the stromal/structural compartment through growth factor supply, as well as maintaining T cells and acting a rich source of IL-10. Although nerve- and blood vessel (BV)-associated IntMϕs have been described, whether these represent obligate niches is under debate.

It is also important to note that due to the overlapping expression of CD11c, CD11b and SiglecF by other myeloid cells, in particular CD11c^+^ dendritic cells (DCs) and SiglecF^+^CD11b^+^ eosinophils, expression of these surface markers alone is not sufficient for the characterisation of murine lung macrophages. Instead, a more rigorous approach is needed to define bona fide murine pulmonary macrophage subsets, with a growing consensus that the optimal strategy is by their co-expression of the high affinity FcγR1 (CD64) and Mer-tyrosine kinase (MerTK), a key efferocytic receptor^12,15,20^. In health, AlvMϕs and IntMϕs can then be identified amongst the CD64^+^MerTK^+^ fraction by their distinct CD11c/CD11b profiles and other phenotypic traits (Fig. 1). As discussed below, distinction between these anatomically distinct cells becomes less apparent when homeostasis is perturbed. Moreover, recent work has shown that some DCs can acquire expression of CD64 in certain contexts^21^, emphasising the need for multi-parameter analysis when characterising these cells. Notably, neither AlvMϕs nor IntMϕs can be defined using the M1/M2 nomenclature system. Indeed, in the healthy lung both populations co-express markers historically considered “M1” and “M2” specific^14,15^. For instance, murine AlvMϕs constitutively co-express CD11c and Ym-1 (encoded by *Chil3*) which have been used by some as defining features of so-called “M1” and “M2” macrophages^22,23^. However, Ym-1 expression by homeostatic AlvMϕs is independent of IL-4–IL-4R signalling^15^, the axis controlling “alternative” activation of macrophages, and CD11c expression is independent of exposure to microbial products or inflammatory cytokines thought to drive so-called “M1” polarisation^24,25^. This highlights the inadequate nature of the M1/M2 nomenclature in defining macrophages in vivo and that as a field we need to abandon using it^26^.

Recent studies employing scRNA-seq have begun to reveal additional heterogeneity in the murine pulmonary macrophage compartment. Whereas AlvMϕs appear to be relatively homogeneous^6^, the IntMϕ compartment harbours at least two distinct subsets defined by their expression of CD206 (mannose receptor)^14,18,27^. The anatomical locale in which each of these subsets are found remains contentious. CD206^−^ IntMϕs that mostly express MHCII^+^ have been suggested to be enriched in the interstitial tissue adjacent to the alveoli, whereas their CD206^+^ MHCII^−^ counterparts appear more numerous in the interstitium surrounding the bronchi^18^. Multi-parameter fluorescence microscopy has also started to elucidate the nature of the niche in which these subsets may exist. For instance, CD206^–^MHCII^+^ IntMϕs can be found to interact with nerves, whereas CD206^+^ MHCII^–^ cells may occupy a perivascular niche^27^. Transcriptional profiling has revealed other useful markers for the identification of these subsets, including Lyve-1, folate receptor beta (FRβ) and CD36, which appear to be more highly expressed by perivascular CD206^+^ MHCII^–^ macrophages. Work by the Khanna group identified a population of CD169-expressing IntMϕs^28^, although it seems these largely overlap transcriptionally with the CD206^–^MHCII^+^ IntMϕs identified by others. CX3CR1 has also been suggested as a defining marker of these subsets^27^, although other studies have shown that CX3CR1 is expressed equally across these subsets^14,18,29^. Thus, while it is clear that the murine IntMϕ compartment is heterogeneous, consensus is yet to be reached on the best strategy to define these cells.

The identity of macrophage subsets in the human lung has also become clearer recently. Human AMϕs express high levels of HLA-DR, CD11b and CD206 together with CD169 and MARCO^7,13,30^. CD163 appears to define two subsets of AMϕ in lung tissue from humans and non-human primates. However, whereas CD163^hi^ AMϕs are abundant in BALF, CD163^lo^ AMϕs are relatively rare^13^, questioning whether they truly represent AlvMϕs. Importantly, while SiglecF has become somewhat of a de facto marker of murine AlvMϕs, its human paralog, Siglec8, is absent from AMϕs in man^13^. Despite only partial conservation in terms of phenotype between mice and humans, recent transcriptional profiling suggests that these cells share a core gene signature, including expression of *PPARG, FABP4, FFAR4, FN1*^7,31–34^ (Fig. 1). The phenotypic and transcriptional identity of human IntMϕs during health is limited by access to healthy lung tissue. Nevertheless, they appear to be defined as HLA-DR^+^CD11b^+^CD36^+^ cells lacking CD169 expression^13^, a phenotype that has recently been confirmed using humanised mice^35^. Moreover, heterogeneity similar to that seen in mice is likely to exist amongst human IntMϕs^27^, with one study suggesting the intensity of HLA-DR and CD36 a defining characteristic^7^.

Box 1 What’s in a name? In order to discuss pulmonary macrophage subsets, some clarity on nomenclature is needed. Historically, macrophages found in the airways/bronchoalveolar lavage fluid (BALF) of naïve mice have been termed ‘alveolar macrophages’, which express high levels of CD11c, SiglecF and CD169 while lacking CD11b expression. These airway/alveolar macrophages are phenotypically and transcriptionally distinct from those found in the lung tissue, which express CD11b and variable levels of CD11c, but lack SiglecF expression and are often termed as ‘lung tissue’, ‘interstitial’ or ‘parenchymal’ macrophages. It is now clear that, during inflammation, macrophages with a tissue/interstitial phenotype can be readily found in the airways/BALF. Thus, for clarity, in this review we will use ‘alveolar’ only to refer to airway macrophages (AMϕs) that are CD11c^hi^SiglecF^+^ (AlvMϕs), and ‘interstitial’ to refer to CD11b^+^SiglecF^–^ lung tissue macrophages (IntMϕs). We will use the term monocyte-derived AMϕs to refer to macrophages in the BALF that have been shown to arise in the context of inflammation and exhibit some, but not all, features of bona fide AlvMϕs. We will use the term ‘ex-interstitial’ (ex-IntMϕ) to try to make clear when we are referring to cases where macrophages with this phenotype are found in the airways. Since it is currently impossible to distinguish tissue vs airway origins of human pulmonary macrophages, we will simply refer to cells obtained by BAL as airway macrophages (AMϕs) and those obtained from human lung tissue as tissue macrophages (tissue Mϕs).

Box 2 Key Challenges

1. Establish “signatures” to faithfully distinguish human AlvMϕs from IntMϕs and ex-IntMϕs
2. Develop methods of lineage tracing in human lung macrophage subsets
3. Reduce reliance on in vitro functional assays of questionable relevance and develop models/systems/approaches that better represent the lung environment
4. Reach consensus on the heterogeneity, function and spatial distribution of IntMϕs
5. Develop novel transgenic murine models to selectively target AlvMϕs vs IntMϕs vs ex-IntMϕs and understand the molecular levers that control their differentiation
6. Transition to using mouse models with microbial exposure/status more aligned with that encountered by humans.

## Macrophage function in health

As immunologists, we tend to consider the primary function of macrophages is to act as the first line of defence against pathogens. Indeed, the positioning of macrophages in the airways means that they will be one of the first cells to encounter air-borne pathogens and altering the ability of airway macrophages to detect, engulf and kill respiratory pathogens leads to increased susceptibility to a variety of bacteria, viruses and fungi^36–41^. However, as described below, to avoid excessive and potentially harmful pro-inflammatory responses being mounted against environmental antigens encountered by airway macrophages, these cells are held in a state of relative hyporesponsiveness via a range of powerful inhibitory mechanisms^42^. In the absence of infection, the principal role of airway macrophages is the regulation of pulmonary surfactant, the lipid-protein complex produced by the respiratory epithelium to lubricate the lungs and allow frictionless expansion/contraction. Consistently, the AlvMϕ transcriptional signature is dominated by genes associated with lipid metabolism in mouse and man^7,43,44^. Macrophages are indispensable for this function, as spontaneous pulmonary alveolar proteinosis (PAP) develops in mice and humans with absent or dysfunctional AlvMϕs^45–50^. Likewise, dead, dying and senescent cells accumulate in the absence of functional airway macrophages, demonstrating their key efferocytic role^43^. Airway macrophages may also maintain the integrity and responsiveness of the respiratory epithelium. For instance, production of immunoregulatory cytokines, such as TGFβ and IL-10, may modify epithelial cell function through regulation of ion and fluid transport^42,51^ and AlvMϕ-derived fibronectin may act as a proliferative factor for airway epithelial cells^52^. Suppressor of cytokine signalling (SOCS)-containing vesicles released from AlvMϕs may regulate the responsiveness of the epithelium to e.g., TLR ligands^53,54^.

Compared with AlvMϕs, the mechanisms of regulation and homeostatic functions of IntMϕs are poorly understood^55^, although it is clear that all murine IntMϕ subsets are avidly phagocytic and can capture *E.coli* bioparticles in vivo^14,18^, suggesting they may act as a second line of defence should the epithelial barrier be breached. Their constitutive production of IL-10 under normal physiological conditions, in both mouse and man^18,29,56–58^, suggests an immunoregulatory role. It is likely this involves supporting regulatory T cells locally in the lung parenchyma, given that IntMϕs are thought to be non-migratory^59^. However, IntMϕs may also alter T cell responses indirectly through IL-10-dependent modulation of DC migration and priming activity^56^.

Exposure to bacterial CpG DNA leads to IntMϕ expansion and augmentation of IL-10 production^29^, suggesting these cells may be inherently anti-inflammatory. Indeed, *Cx3cr1*-mediated deletion of *Il10* leads to increases susceptibility to allergic asthma in mice^60^. Production of platelet-derived growth factor (PDGF) by IntMϕs suggests they may also support fibroblast and epithelial homeostasis^61^. Finally, given their occupation of distinct anatomical niches, it is intriguing to speculate they may differentially contribute to nerve and vascular endothelial cell homeostasis, similar to their counterparts in the gut wall^62^.

## Lung macrophage ontogeny

### Macrophage origins in health

Historically, macrophages were thought to be part of a linear mononuclear phagocyte system where tissue macrophages were continually replaced by blood monocytes, which themselves are replaced by dedicated bone marrow progenitors^63^. However, over the last 10 years there has been a conceptual revolution in our understanding of macrophage ontogeny with the discovery that many tissue macrophages derive from embryonic progenitors and maintain themselves autonomously through in situ self-renewal^64–74^. While some older studies had demonstrated the ability of macrophages to self-renew^75^, it is the development of elegant lineage tracing models that has led to major advances in our understanding of macrophage origins. For instance, genetic fate mapping using mice with tamoxifen-inducible Cre recombinase under the control of the *Csf1r, Runx1, Cx3cr1* or *Tie2* promoters has shown that brain microglia derive from yolk sac progenitors and require little, if any, contribution from blood monocytes across the life of an animal^64,65,67,69,76^. Using similar systems, it was shown that yolk sac progenitors contribute minimally to lung AlvMϕs^77^. Instead, tracing of foetal and adult haematopoiesis using *Flt3*^Cre^ mice shows haematopoietic stem cell (HSC)-derived cells make a major contribution to AlvMϕs^65,68,74^. This, combined with the fact that AlvMϕs develop within the first few days of life in mice and humans in parallel with alveolisation of the lung^13,78,79^, led to the idea that they derive predominantly from foetal monocytes. That AlvMϕs are unaffected in adult monocytopenic *Ccr2*^–/–^ mice and show little exchange in the context of parabiosis or tissue protected bone marrow chimeric mice supported the notion that these cells self-maintain throughout adult life in the absence of inflammation or infection^68,78^. Moreover, analysis of AMϕ longevity in the human context supported these observations in mice. By analysing macrophages obtained by transbronchial biopsies of recipients of sex mismatched lung transplants in a longitudinal manner, Eguíluz-Garcia *et al*. showed that the majority of AMϕs remain of donor origin in this setting, suggesting human AMϕs maintain themselves autonomously in situ^80^, a finding supported by an independent study analysing AMϕs obtained by bronchoalveolar lavage^81^.

**Fig. 2:**
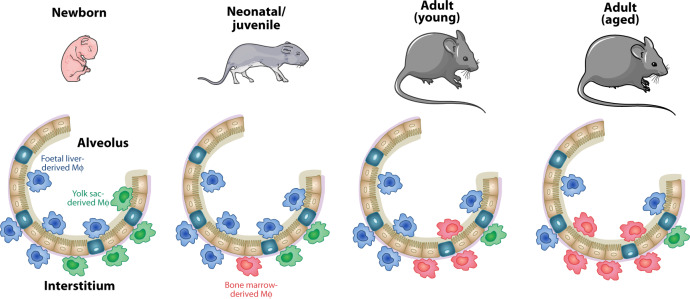
Pulmonary macrophage ontogeny during health. The contribution of distinct progenitors to the pulmonary macrophage compartments is highly dynamic and alters with age. During embryonic development (in mice) yolk sac-derived macrophages colonise the lung and these remain present at birth. However, these are outnumbered by foetal liver-derived progenitors that enter the lung prior to birth, some of which move into the airways upon alveolarization within the first days of life. During the neonatal period, where there is massive tissue growth, all macrophages show high levels of proliferation to occupy the newly created niches. This is sufficient to expand the AlvMϕ compartment with little, if any, contribution from bone marrow-derived monocytes. However, recent work has suggested that during adulthood under homeostatic conditions AlvMϕs are replenished, albeit at low rates, by bone marrow-derived, CCR2-dependent monocytes. These monocytes replace IntMϕs at a higher rate, although in the unperturbed lung, the IntMϕ compartment likely contains macrophages derived from the yolk sac, foetal liver and bone marrow, with the latter dominating numerically.

However, several recent studies have started to challenge this model. First, longitudinal analysis of *Flt3*^Cre^-*Rosa26*^LSL-YFP^ reporter mice showed increases in labelling of AlvMϕs over time, indicative of age-dependent contribution of HSC-derived cells to the AlvMϕpool, a phenomenon not seen in brain microglia^65^. This highlights the need for longitudinal analysis when considering macrophage dynamics, something that was not always performed in early lineage tracing studies^64,74^. Indeed, longitudinal analysis of *Ms4a3*^Cre^ reporter mice, which allow tracing of all cells deriving from bone marrow granulocyte-monocyte progenitors (GMPs), supports the idea that AlvMϕs require replenishment from bone marrow-derived monocytes over the life course^71^ (Fig. 2). These data are consistent with recent work assessing AlvMϕ turnover using so-called ‘MISTRG’ humanised mice, which have genes encoding human M-CSF (also known as CSF-1), GM-CSF (also known as CSF-2), IL-3 and thrombopoietin ‘knocked-in’ to their respective mouse loci to support human myeloid cell development, as well as a transgene encoding human SIRPα to prevent engulfment and destruction of human cells^35,82^. Moreover, the idea of AlvMϕ replenishment by monocytes is supported by a recent study using scRNA-seq to determine AMϕ longevity in the context of sex-mismatch lung transplants where the majority of donor AMϕs appear to be replaced by recipient cells^83^. Why different studies using transplanted tissue reach discordant conclusions is unclear, but could reflect differences in methodologies used, for instance scRNA-seq versus fluorescence in situ hybridisation (FISH) for X/Y chromosomes, or the degree of injury caused by transplantation-related ischaemia and reperfusion. Clearly further work is warranted to clarify the dominant replenishment mechanisms underlying the homeostatic maintenance of AlvMϕs.

The developmental origin of IntMϕs has started to be unravelled in the past few years, although these cells have attracted much less attention than their AlvMϕ counterparts. Genetic fate mapping indicates an initial contribution of yolk sac progenitors to the IntM pool^65,77^, but these appear to be largely replaced, first by foetal liver-derived macrophages and then by HSC-derived macrophages during the early post-natal period^27,65^ (Fig. 2). CCR2-dependent bone marrow-derived cells continue to replenish IntMϕs during adulthood, albeit at a low rate, and despite the heterogeneity described above, IntMϕ subsets appear to display similar replenishment kinetics^27,84^. Notably, despite clear evidence of progressive replenishment by monocytes, intact *Ccr2*^*–/–*^ mice have normal numbers of IntMϕs^18,29^, serving as a cautionary note that *Ccr2*^–/–^ mice cannot be used in isolation to determine the contribution of monocytes to tissue macrophage pools, at least in health. This suggests potential redundancy between chemokine receptors involved in monocyte navigation^85^ and/or that compensatory mechanisms maintain macrophages in the context of monocytopenia. Indeed, both AlvMϕs and IntMϕs can and do proliferate under normal physiological conditions^15,68^. While this appears to be insufficient to maintain these populations, it may be sufficient in the absence of monocytes. It is also important to appreciate that self-renewal and derivation from monocytes are not mutually exclusive mechanisms of macrophage maintenance and, in certain contexts, monocyte-derived macrophages proliferate more readily than their embryonic counterparts^73,86,87^. Replenishment by monocytes has been proposed to arise in response to niche availability^88^, although the factors that govern ‘availability’ remain poorly understood and it is unclear if this differs in distinct subanatomical niches within the lung. Alternatively, there is evidence that different precursors have distinct metabolic states that may determine their long-term persistence in the lung, suggesting that cell-intrinsic properties may also govern replenishment kinetics^89^.

### Macrophage origin following injury, inflammation or infections

**Fig. 3:**
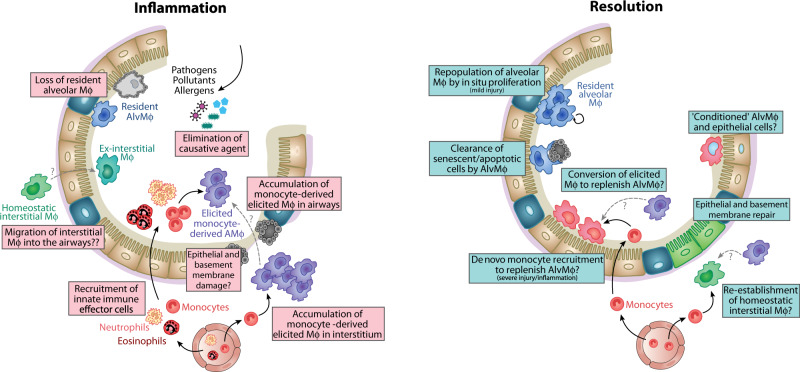
Pulmonary macrophage dynamics during inflammation and resolution. In inflammation caused by agents such as pathogens, pollutants or allergens, most resident AlvMϕs are lost and replaced by monocyte-derived Mϕs and perhaps ex-IntMϕs. This occurs in parallel to accumulation of other inflammatory cells such as neutrophils and eosinophils, recruitment of which to the airways is facilitated by chemokines and disrupted barrier integrity. During resolution of the damage caused by acute inflammation, and/or in the face of chronic low-level inflammation, residual AlvMϕs can self-renew through proliferation, clear up dying or dysfunctional cells in the airways, as well as be replenished through conversion of monocyte-derived macrophages and ex-IntMϕs which are transcriptionally, epigenetically and functionally conditioned by the airway environment to take on AlvMϕ identity.

The mechanisms that govern macrophage expansion or repopulation following injury, infection or inflammation appear to be dependent on the nature of the insult. Acute inflammation or severe infection often leads to loss of tissue resident macrophages, a phenomenon described as the ‘macrophage disappearance reaction’ (Fig. 3). For instance, sterile inflammation induced by instillation of LPS in mice leads to a transient loss of AlvMϕs and expansion of IntMϕs in the tissue (unpublished observations). IntMϕ expansion is impeded by *Ccr2* deficiency^29^, suggesting a major role for monocyte recruitment in this process. In contrast, AlvMϕ repopulation during inflammation resolution appears to rely exclusively on local proliferation^71^. In contrast, following a more substantial inflammatory insult, such as that induced by administration of bleomycin or silica to model lung fibrosis or infection with influenza, leads to replacement of resident AlvMϕs with monocyte-derived cells^90,91^. Consistent with this, monocyte-derived cells come to dominate the AMϕ compartment in individuals with severe Sars-CoV-2 infection^5^. Whether this reflects direct effects of severe inflammation on the self-renewal capacity of AlvMϕs, or if severe inflammation leads to structural alterations, such as breakdown of basement membrane and epithelial integrity, is unknown. While classical monocytes can enter the airways in response to injury or infection, there is evidence that monocyte-derived, elicited macrophages in the interstitium may also transition to the airways. Whether these alternative differentiation routes influence the fate and function of these cells is currently unclear.

## Environmental imprinting of lung macrophages

The diversity of macrophages within the lung results from their plasticity and ability to respond to local environmental cues. In this regard, the pulmonary environment is unusual, even in comparison to other barrier sites, in terms of the wide range of environmental features it presents that can influence immune cell recruitment, activation and function, and that will have a particular impact on lumen-dwelling AlvMϕs. These include the unique makeup of the airway fluids (predominantly composed of surfactant and mucins), commensals (bacteria, viruses and fungi) and nutrient levels (e.g., both host and microbial derived metabolites), all of which can change markedly during inflammation.

As well as acting as a lubricant, pulmonary surfactant constituents can also influence AlvMϕ behaviour. For instance, in the absence of surfactant protein D (SP-D) AlvMϕs display an unusual CD11b^hi^ phenotype and constitutively produce TNFα^92^. While phenotypic alterations are not evident in AlvMϕs from naïve SP-A deficient mice^93^, SP-A may amplify IL-4Rα-mediated AlvMϕ activation while regulating their responsiveness to exogenous stimulation through direct interactions with Toll-like receptor 4 (TLR4) and MD-2^93–95^. As collectins, surfactant conformation can dictate their function in a context-dependent manner, with structural changes altering their binding to targets and in doing so conferring pro- vs anti-inflammatory ability^96^.

The other major constituent of pulmonary fluids is mucus, as vital for lubricating the airways as surfactant, and also for entrapment and cilliary clearance of inhaled particles and microbes^97^. Additionally, mucus can be important for control of bacterial infection, regulation of hydration, resolution of inflammation, and modulation of immune and epithelial cell function^98,99^. The main mucins expressed in the lung are Muc5b and Muc5ac, with Muc5b being essential for maintenance of healthy airways^97,99^, while Muc5ac is up-regulated during inflammation^100–102^. Each of these mucins has different properties that determine their dominant function. Mucus also provides a home for commensal lung microbes^97^, which can exert a dramatic effect over airway function, for example through invasion/colonisation, consumption of nutrients, and production of metabolites – all of which can influence pulmonary inflammation and macrophage activation and function. Although most is currently known about bacteria and their products in this context, more widespread metagenomic approaches will increase our understanding of the abundance and diversity of bacterial, viral and fungal commensals in the lung, and how this changes during inflammation and disease.

In terms of nutrients, the airways present AlvMϕs with one of the lowest glucose environments in the body, a tissue adaptation that has likely evolved to prevent outgrowth of glucose-hungry opportunistic bacteria^103,104^. Indeed, epithelial cells lining the lung express high levels of glucose transporters apically^105^, with which they can rapidly and efficiently reduce glucose levels in the airways, with the blood and tissues underlying the epithelium possessing over twelve times the levels of glucose than are found in the airway fluids^103,105^. This low-glucose environment, coupled with abundant lipid-rich surfactant, likely plays a central role in governing AlvMϕ metabolism and function. Indeed, it is now clear that metabolism is a central factor in governing macrophage activation and function, with the general principle being that glycolysis may be more associated with ‘type 1’ macrophages, while lipid metabolism tends to be more typical of ‘type 2’ macrophages (reviewed by^106,107^). However, the majority of the work that has established this paradigm has relied on in vitro assessment of bone marrow or monocyte-derived macrophages, with much less understanding of how tissue environments influence macrophage metabolism in vivo. In the context of the lung, we and others have shown that AlvMϕs display a distinctive metabolic profile, expressing elevated levels of genes associated with lipid metabolism and peroxisome proliferator-activated receptor gamma (PPARγ), and reduced expression of genes associated with glycolysis^15,20^. Further, we have shown that glycolysis is a key determinant of AlvMϕ activation and function in type 2 inflammation^15^, suggesting that glycolytic ability, and availability of glucose, may be centrally involved in enabling AlvMϕ activation and function in the airways.

During inflammation, with compromised epithelial integrity, the balance of airway nutrients can change markedly, with reports of elevated airway glucose evident in a range of disease settings, including COPD, cystic fibrosis and asthma^108–111^. This may be particularly relevant in chronic conditions where metabolite balance in the airways can be modified long-term, with consequent long-term alteration of AlvMϕ metabolism and function. In more acute settings, such mechanisms may enable a window of opportunity for glycolytic ‘boosting’ of AlvMϕ function that will reduce as the epithelium heals and metabolite levels recalibrate, in essence providing a metabolic rheostat for fine-tuning of AlvMϕ activation and function directly linked to how local substrate levels change in line with levels of tissue damage vs resolution.

In contrast to AlvMϕs, less is currently known about metabolic control of IntMϕ activation and function. IntMϕs residing in the more nutrient-rich environment of the lung tissue appear much more glycolytically active than their AlvMϕ counterparts, and are consequently more effective at producing the reactive oxygen species necessary for killing intracellular bacteria such as *Mycobacterium tuberculosis* ^20,112^. In this way, IntMϕs may be less susceptible to substrate-related functional control than AlvMϕs, though this likely changes if IntMϕs migrate into the airways in the context of inflammation as they adapt to this new environment. Indeed, a key outstanding question is to what extent does the metabolic status of monocyte-derived macrophages play in their differentiation to AlvMϕs during and following an inflammatory insult. It is important, however, to remember that the methods to extract tissue macrophages can have profound effects on their biology, including their transcriptional and metabolic status. Given that IntMϕs are embedded in the tissue, it is plausible that some of the differences seen in their metabolic profile may reflect their response to extraction.

As described above, there is continual bi-directional crosstalk between macrophages and the structural/stromal cells that comprise their niche, and this crosstalk leads to niche-specific outcomes for macrophage recruitment, differentiation and function. Removing macrophages from their niche leads to phenotypic and transcriptional changes, directly demonstrating the need for continual crosstalk with structural cells^3^. In the airways, AlvMϕs are highly dependent on GM-CSF for their development and maintenance^78,113–117^, consistent with their high expression of GM-CSF receptor. Consequently, disruption to the GM-CSF-GM-CSFR axis leads to defective AlvMϕ differentiation and the development of PAP in both mice and humans^45–50^. Recent work using GM-CSF reporter mice and cell-specific deletion of GM-CSF has identified alveolar type 2 epithelial cells as the indispensable source of GM-CSF during the pre- and post-natal stages of AlvMϕ development from foetal progenitors^117^. Interestingly, although innate lymphoid cells (ILCs) are major sources of GM-CSF in the steady state lung, haematopoietic deletion of GM-CSF does not affect AlvMϕ development^117^. Moreover, although it has been proposed that GM-CSF may control AlvMϕs indirectly through induction of *Il6, Il13* and *Csf1* expression in lung basophils^118^, genetic depletion of basophils has little, if any, effect on AlvMϕ number or phenotype^117^. Indeed, that CD11c-mediated deletion of *Csf2rb*, which encodes one of the GM-CSFR subunits, or *Stat5*, which lies downstream of GM-CSFR, leads to aborted differentiation of AlvMϕs^119,120^ supports the notion that GM-CSF acts directly on developing AlvMϕs.

As mentioned above, crosstalk between AlvMϕs and alveolar epithelial cells also involves the TGFβ-TGFβR axis. TGFβ is a potent immunomodulatory cytokine which is abundant in the mouse and human lung in health. It is produced in a latent form and must be converted to active TGFβ to have biological effects. In the airways, integrin-mediated activation is thought to be the principal mechanism of TGFβ activation. Specifically, the αvβ6 integrin, which is expressed by alveolar epithelial cells, is crucial for generating active TGFβ^121^. Consequently, genetic disruption of *Itgb6*, which encodes integrin β6, leads to development of emphysema due to excessive production of MMP12 by dysfunctional AlvMϕs; a phenotype that can be rescued by constitutive expression of TGFβ1^121,122^. Human AMϕs are known to have a gene signature consistent with TGFβR signalling^123^ and myeloid-specific deletion of TGFβR in mice leads to aborted AlvMϕ development, demonstrating a need for cell intrinsic TGFβR for this process^124,125^. Interestingly, although many cells can produce TGFβ, macrophages themselves are thought to be an important source^123,124^. Moreover, AlvMϕs can facilitate integrin-mediated release of active TGFβ through production of amphiregulin, at least in the context of helminth infection^126^. Loss of TGFβR signalling leads to reduced expression of GM-CSFR, suggesting cooperation between these factors^124^. However, the indispensable nature of TGFβ in their development makes assessing its role in regulation of AlvMϕ behaviour during homeostasis difficult. Nevertheless, TGFβ is thought to upregulate the inhibitory receptor CD200R1, which is known to be key for maintaining the activation threshold of AlvMϕs^127^. Consistent with this, loss of autocrine TGFβ leads to spontaneous production of pro-inflammatory cytokines and chemokines by AlvMϕs^123^.

Both GM-CSF and TGFβ induce expression of the transcription factor PPARγ, which is considered the master transcription factor for AlvMϕs^43,124^. Mice with myeloid-specific deletion of *Pparg* also develop PAP, consistent with regulation of molecules involved in lipid catabolism by PPARγ^43,128,129^. Given that PPARγ is expressed by macrophages in other tissues, including splenic red pulp macrophages and macrophages of the erythroblastic islands in the bone marrow^129,130^, until recently, it remained unclear how specificity was conferred to AlvMϕs. We recently uncovered the transcription factor early growth response 2 (EGR2) as a key evolutionarily conserved regulator of AlvMϕ differentiation downstream of PPARγ in the lung but not spleen^125^. Interestingly, mice with *Egr2* deficient AlvMϕs and individuals with mutations in *EGR2* do not appear to develop spontaneous PAP^125,131^, demonstrating that PPARγ must cooperate with other transcriptional regulators to regulate distinct aspects of AlvMϕ biology. For instance, EGR2 appears particularly important for regulating expression of adhesion molecules, chemotactic machinery and apparatus for the detection and elimination of respiratory pathogens^125^. EGR2 appears to maintain expression of CCAAT/enhancer-binding protein beta (C/EBPβ), which has been implicated in AlvMϕ differentiation^132^. The transcription factors Bhlhe40 and Bhlhe41 have also been shown to control the phenotypic identity and proliferative capacity of AlvMϕs, and seem to rely on TGFβR signalling in a PPARγ-independent manner^133^. The histone deacetylase (HDAC) sirtuin 1 (SIRT1) also plays a key role in regulating the proliferative activity of AlvMϕs^134^. Finally, Bach2 (B lymphoid transcriptional repressor BTB and CNC homology 2) has been shown to be essential for surfactant regulation by AlvMϕs^135^. Thus, while much progress has been made in understanding the transcriptional control of lung macrophages, if and how these transcriptional regulators interact or cooperate to control the discrete molecular programmes required for homeostatic function of AlvMϕs is only starting to be understood and warrants further study using state-of-the-art technologies.

The environmental control of IntMϕs is much less well understood. Despite high expression of CX3CR1 by at least some IntMϕs, their survival, phenotype and proliferative capacity is unaffected by *Cx3cr1* deficiency^14^. Unlike their alveolar counterparts, IntMϕs rely on signalling through CSF1R for their development and maintenance as evidenced by their depletion with anti-CSF1R antibody treatment^28^ and failure to develop from *Csf1r*^–/–^ precursors in a competitive bone marrow chimera setting^136^. The relative role of the ligands for the CSF1R, M-CSF and IL-34, has not been examined exhaustively. For instance, although cDC2s are reported to be affected in *Il34*^LacZ/LacZ^ mice^137^, these cells were simply defined as CD11c^+^CD11b^+^ non-AlvMϕs and it is highly likely this compartment contains both IntMϕs and cDC2s. Similarly, analysis of *Csf1*^op/op^ mice, which have a naturally occurring inactivating mutation in the *Csf1* gene, has shown an effect on the abundance of CD169^+^ but not CD169^–^ IntMϕs^28^, suggesting differential reliance on M-CSF by discrete IntMϕ subsets. Application of novel reporter and conditional “KO” mice, such as those used to identify the cellular sources of M-CSF in the lymph node and spleen^138,139^, should help discern the relative roles and cellular origin of M-CSF and IL-34 in regulating survival and differentiation of IntMϕ subsets.

The downstream molecular pathways that govern IntMϕ differentiation remain largely elusive. Although expression of *Maf, Mafb, Irf5, Jun* and *Atf3* have been identified through scRNA-seq studies as highly expressed by murine IntMϕs^6,125^, if and how these transcription factors control their differentiation remains unexplored. Furthermore, given that some of these (e.g., IRF5) have been implicated in AlvMϕ homeostasis^140^, high expression does not always equate to specificity. Importantly, although dispensable for the phenotypic identity and survival of IntMϕs, β-catenin signalling has recently been implicated in the control of the metabolic profile of IntMϕs, in response to the Wnt family molecule Rspondin3 derived from pulmonary endothelial cells^141^. IntMϕs are intimately associated with extracellular matrix and interaction with collagen via the collagen receptor, LAIR1, appears to alter the composition of the IntMϕ pool. Notably, LAIR1 appears to regulate CSF1R expression^142^ and therefore interactions with the ECM may regulate macrophage longevity, although this remains to be tested experimentally.

Thus, it is clear that the lung environment exerts multiple layers of control over macrophage development, activation and function in health and during inflammation. This highlights the current over-reliance on in vitro methods to research lung macrophages, particularly for human research, which are likely of questionable relevance. Innovative new approaches are needed that better reflect the lung environment, such as ‘lung on a chip’^143–146^, organoids^147,148^, and maintenance of whole lung tissue ex vivo.

## Macrophages in pulmonary fibrosis

Despite their key roles in lung homeostasis, macrophages are implicated in the pathogenesis of many chronic lung pathologies, including pulmonary fibrosis (PF). PF is a common feature of a group of conditions known as interstitial lung diseases (ILDs), where excessive ECM deposition leads to irreversible scarring of the lung (reviewed by^149,150^). In many cases the cause of pulmonary fibrosis is not identified (idiopathic pulmonary fibrosis (IPF)), whereas in others it can be attributed to exposure certain occupational substances (e.g., asbestos, silica) or drugs (e.g., bleomycin, methotrexate). Moreover, there are indications that following severe coronavirus disease 19 (COVID-19), certain individuals develop pulmonary fibrosis^151^, although whether this results in permanent, irreversible scarring is still being understood.

While the prevailing school of thought is that PF arises from ineffective repair of airway epithelium following repetitive injury, there is now compelling evidence that macrophages contribute to PF pathology^152^ and that targeting macrophages could be beneficial in human disease^153^. First, there is vast macrophage accumulation in the lung parenchyma during PF and experimental fibrosis where they co-localise with collagen-producing myofibroblasts and support their proliferation and function through production of PDGFα, PDGFβ, TGFβ1 and Galectin-3^30,154–157^. Macrophages have also been shown to be rich sources of osteopontin (encoded by *Spp1/SPP1*)^32,90,155,158^, which has long been established as a pro-fibrotic mediator, in part through activation of TGFβ1^158^. Osteopontin-producing macrophages are found in the airways and parenchyma of IPF individuals^159^, although highest expression is attributed to *MAFB*^+^*PPARG*^–^ macrophages, which most likely represent IntMϕs^32^. Moreover, high expression of inhibitors of collagenolytic enzymes, including tissue inhibitor of metalloproteinases 1 (TIMP1) and TIMP2 is a feature of fibrosis-associated macrophages^154^. Studies in mice have shown macrophages to be able to produce certain collagens (e.g., collagen VI), and collagen VI deficiency limited to the haematopoietic compartment ameliorates experimental bleomycin-induced fibrosis^160^. Interestingly, however, elevated expression of matrix metalloproteinases, such as MMP-9, MMP-12 and MMP-14, also defines fibrosis-associated macrophages across species. Thus, the relative contribution of collagen production versus collagen clearance remains poorly understood.

Macrophage accumulation results, at least in part, from de novo recruitment of CCR2^+^ monocytes in both experimental models and human PF^161^. The presence of CCR2^+^ monocytes and their macrophage progeny correlates with the presence of fibrotic tissue in mouse and man^161^, and bleomycin-induced experimental fibrosis can be exacerbated by adoptive transfer of classical monocytes^162^. Consistent with this, experimental fibrosis is blunted in monocytopenic *Ccr2* deficient mice^161,163,164^, by neutralisation of the CSF1-CSF1R axis^154^ or by rendering monocyte-derived macrophages susceptible to apoptosis^165^. Moreover, one of the few treatments for PF, pirfenidone, has recently been shown to reduce accumulation of CCR2^+^ monocytes in bleomycin-induced experimental fibrosis^161^. However, the Akira group has suggested that developmentally distinct, pro-fibrotic monocytes termed “segregated-nucleus-containing atypical monocytes” (SatM) arise in the context of experimental lung fibrosis and are responsible for driving disease^166^. So-called ‘SatM’ appear to depend on the transcription factor C/EBPβ, but derive from FcεR1^+^ granulocyte/macrophage progenitors (GMPs) and not macrophage/DC progenitors (MDPs)^166^. Indeed, work since has described distinct pathways to generate monocytes from GMPs and MDPs in health and following infection^167^. How these SatM relate to the CCR2-dependent monocytes described in other studies remains unclear.

Whether pro-fibrotic macrophages are limited to the lung parenchyma or if monocyte-derived AMϕs also contribute to fibrosis is still under debate^90,125,154,156^. In support of the latter, sustained epithelial injury is a feature of human PF^155,168^ and reducing epithelial damage through administration of a specific inhibitor of sphingosine kinase 1, which is elevated in IPF lungs, reduces experimental fibrosis, at least in part by reducing recruitment of fibrogenic monocytes^169^. Attributing key pathogenic roles to macrophage subsets is made difficult by the breakdown in clear phenotypic boundaries between parenchymal and AlvMϕs in the context of inflammation and fibrosis, and by the fact that, at least some, IntMϕs may differentiate into AlvMϕs during lung repair^125,156^. PF incidence and severity positively correlates with age^170–172^ and given monocytes may progressive replace embryonically-derived AlvMϕs with age, it is intriguing to speculate that these two phenomena could be related. In addition to blood-derived monocytes, it is plausible that macrophages in the pleural cavity may contribute to the pro-fibrotic pool of macrophages in the lung. Consistent with this notion, fibrosis in IPF patients is often concentrated in the subpleural region^150^ and there is transcriptional similarity between pleural and MHCII^–^ IntMϕs in mice^27,173^. Moreover, it’s been suggested serous cavity macrophages may contribute to tissue repair in neighbouring solid organs following injury in mice^174,175^. However, elegant intersectional genetics and a combination of injury models have shown that, while pleural GATA6+ macrophages may accumulate on the pleural membrane, they do not migrate deep into the lung parenchyma nor are they essential for fibrogenesis or resolution^176^.

Why macrophages become excessively pro-fibrotic is only starting to be understood, although, again, this probably reflects their plasticity. IL-4, IL-13, IL-33 and TGFβ have all been implicated in altering macrophage behaviour in PF (reviewed by^152^), but the relative and combinatorial roles of these factors is poorly understood. Recently, overactive Notch signalling has been implicated in the pro-fibrotic behaviour of macrophages, as deletion of RBPJ reduces fibrosis in mice through abrogating TGFβ production by Ly6C^hi^MHCII^+^ monocyte-derived macrophages^177^. Whether these effects are attributable to airway or IntMϕs is difficult to discern in this study^177^. TGFβ is of particular interest given its long-standing role in tissue fibrosis^178^. Recent work has uncovered discrete functions of TGFβ isoforms in the fibrotic process^179^. However, if and how excessive TGFβ isoforms influence macrophage function in the fibrotic niche in vivo has not been tested directly.

Like in many pathologies, the role of macrophages in lung fibrosis is very much context dependent. The self-resolving nature of some experimental models allows macrophage dynamics and behaviour to be assessed during fibrosis regression and resolution, something that cannot be gleaned from human disease. This has revealed that severe lung injury leads to almost complete replacement of embryonically derived AlvMϕs with monocyte-derived cells^90,125^. We have recently shown that this process is highly dependent on the transcription factor EGR2 and that EGR2-dependent monocyte-derived AMϕs are indispensable for resolution of fibrosis and restoration of airway homeostasis^125^, findings consistent with older non-specific depletion studies in mice^162^. There is evidence this may involve direct clearance of collagen by (monocyte-derived) AMϕs. For instance, genetic ablation of milk fate globule epidermal growth factor 8 (Mfge8), a receptor typically associated with efferocytosis, leads to failed collagen clearance following bleomycin-induced injury^180^. Moreover, macrophage-derived ApoE may facilitate binding and targeting of type I collagen for phagocytosis via the low-density lipoprotein receptor-related protein 1 (LRP1)^181^. Pro-resolution roles of monocyte-derived AMϕs are also seen following influenza infection and their absence can result in the development of fibrosis, at least in mice^182^. Such functions may include metabolic rewiring of monocyte-derived macrophages as deletion of *Acod1*, the enzyme required for generation of the metabolite itaconate, leads to persistent fibrosis^183^. Thus, while generally considered as key pro-fibrotic cellular players, monocyte-derived AMϕs appear to have a crucial role in re-establishing lung homeostasis and may hold great promise for therapeutic targeting to promote fibrosis regression and lung repair.

## Conclusion

The past few years have seen a leap forwards in our understanding of pulmonary macrophage development, heterogeneity and function, and how environmental features of the lung can exert a dramatic influence over these processes in both disease and in health. A major challenge for the coming years is to develop much greater clarity on how different inflammatory conditions alter pulmonary macrophage subset diversity and function, in particular in the context of human disease, to identify core mechanisms that might enable development of the targeted therapeutics of the future.
